# Probing correlated states with plasmons

**DOI:** 10.1126/sciadv.adg3262

**Published:** 2023-04-26

**Authors:** Michał Papaj, Cyprian Lewandowski

**Affiliations:** ^1^ Department of Physics, University of California, Berkeley, CA 94720, USA.; ^2^ National High Magnetic Field Laboratory, Tallahassee, FL, 32310, USA.; ^3^ Department of Physics, Florida State University, Tallahassee, FL 32306, USA.

## Abstract

Understanding the nature of strongly correlated states in flat-band materials (such as moiré heterostructures) is at the forefront of both experimental and theoretical pursuits. While magnetotransport, scanning probe, and optical techniques are often very successful in investigating the properties of the underlying order, the exact nature of the ground state often remains unknown. Here, we propose to leverage strong light-matter coupling present in the flat-band systems to gain insight through dynamical dielectric response into the structure of the many-body ground state. We argue that because of the enlargement of the effective lattice of the system arising from correlations, conventional long-range plasmon becomes “folded” to yield a multiband plasmon spectrum. We detail several mechanisms through which the structure of the plasmon spectrum and that of the dynamical dielectric response is susceptible to the underlying order, revealing valued insights such as the interaction-driven band gaps, spin-structure, and the order periodicity.

## Teaser

Plasmons enable study of correlated states in flat-band materials.

## INTRODUCTION

The moiré material paradigm of combining two-dimensional (2D) weakly interacting materials to yield strongly interacting systems is at the forefront of the current condensed matter research (*1*–*3*). Moiré systems exhibit a wide range of phenomena ranging from unconventional superconductivity to various interaction-induced (correlated) resistive states (*4*–*19*). Identification of the microscopic nature of the correlated states in the moiré systems is, however, difficult, as it relies on the interpretation of transport behavior or scanning-tunneling microscopy measurements (*20*–*30*). To that end, despite the intense experimental and theoretical efforts, the exact flavor of the ground states is often not certain; thus, new tools to help identify them and complement existing results are in high demand. One such approach can be based on the study of plasmons, the collective charge excitations of interacting electron systems (*31*–*38*), and the overall properties of the system’s dynamical dielectric response.

A defining characteristic of the moiré materials making their dielectric response distinct from that of conventional condensed matter systems is the large effective lattice constant (*a_M_*∼ 10 nm) (*39*–*41*). The large unit cell size makes microscopic variations of the electric fields on the scale of the moiré period a substantial effect, in contrast to the ordinary crystals with lattice constants *a*∼ 0.1 nm, necessitating consideration of local field effects, i.e., treatment of the screening effects accounting for the variation of the electric field within the unit cell (*42*–*44*). This phenomenon gives rise to a dynamical response function matrix ε_**GG**′_(**q**, ω), which not only depends on the frequency ω and momentum **q** inside the Brillouin zone (BZ) but is also labeled by the reciprocal lattice vectors **G**, **G**′ that relate Fourier components of the cell-periodic electric field. Crucially, the dynamical response function matrix contains information about possible plasmon resonances, which are given by the solution of det ε_**GG**′_(**q**, ω) = 0 (*45*–*47*). Because of the matrix structure of the dielectric function, this characteristic equation can, in analogy to the free electron model, yield several branches of collective excitations.

**Fig. 1.**
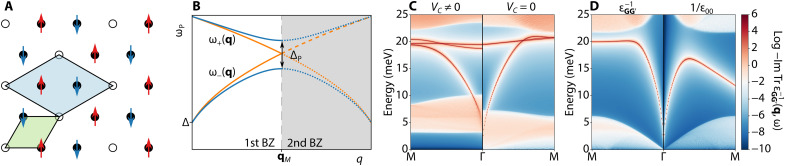
The role of local field effects in moiré materials. (**A**) With a correlated ground state, the moiré unit cell (green) expands to a larger effective cell (blue) that takes into account charge and spin ordering. An example is given for filling ν = 2/3 and AFM state. (**B**) Schematic depiction of plasmon folding when crystal unit cell is extended. The two interband plasmon branches ω_±_(**q**) are given by Eq. 11, with Δ being the correlated gap and Δ_P_ the plasmonic gap as defined in the text. (**C**) The appearance of a correlated state introduces multiple plasmon branches and opens up gaps both between plasmon bands as well as in the particle-hole continuum. (**D**) In the absence of correlated state and unit cell enlargement, the inclusion of local field effects (left panel) does not introduce additional plasmon branches compared to when such effects are neglected (right panel).

In this work, we propose that the structure of the folded plasmon resonances, together with the overall behavior of the dynamical dielectric response, can allow for direct characterization of the microscopic nature of the correlated phases and their underlying ground states. While our findings are general and applicable to any flat-band material, we focus on heterobilayer moiré transition metal dichalcogenides (TMDs) (*12*–*19*), where the difference in lattice constant of the materials in the two layers gives rise to the moiré pattern. Experimentally, these systems exhibit interaction-induced insulating states at fractional and integer fillings consistent with generalized Wigner crystals pinned to the effective moiré lattice sites as demonstrated in Fig. 1A. Theoretically, as attributed to the low-energy band theory with a localized Wannier basis description, these insulating states are qualitatively well described by a Hubbard model (*48*–*51*). Adopting this identification of the underlying competing ground states, we show how the different ground states can result in drastically different dynamical responses, particularly in the plasmon spectrum, thus allowing for identification of such correlated states in the experiments.

## RESULTS

### Dielectric function with local field effects

The formalism for treating local field effects has been introduced in the seminal works (*42*, *43*). Specifically, when calculated within the random phase approximation (RPA), the dielectric function matrix takes the following form (*42*–*44*)

$$\varepsilon_{{GG}^{\prime }}(q,\omega )=\delta_{{GG}^{\prime }}-T_{{GG}^{\prime }}(q,\omega )$$ (1)

$$\begin{array}{c} T_{GG^{\prime }}(q,\omega )=V_{q+G}\sum_{n,m,k}\frac{f_{0}(\varepsilon_{nk})-f_{0}(\varepsilon_{mk+q})}{\omega +i0^{+}+\varepsilon_{nk}-\varepsilon_{mk+q}}\\ \times \eta_{q,G}^{nm}{\left(k\right)}^{*}\eta_{q,G^{\prime }}^{nm}(k) \end{array}$$ (2)

where the Fourier transform of the Coulomb potential is given by $V_{q}=\frac{2\pi e^{2}}{\kappa |q|}$ (with κ being the dielectric constant of the surrounding insulating gate material) and *f*_0_(ε) = [*e*^β(ε − μ)^ + 1]^−1^ (with μ being the chemical potential and β = 1/*k*_B_*T* being the inverse temperature). The state overlap $\eta_{q,G^{\prime }}^{nm}(k)$

$$\eta_{q,G}^{nm}(k)=\frac{1}{\text{Ω}}\int_{\text{Ω}}d^{2}r\;u_{nk}{\left(r\right)}^{†}e^{-iG\cdot r}u_{mk+q}(r)$$ (3)

is evaluated using *u*_*n***k**_(**r**), the cell-periodic part of the Bloch wave function ψ_*n***k**_(**r**) = *u*_*n***k**_(**r**)*e*^*i***k**·**r**^ for an eigenstate from band *n* with a BZ momentum **k** and energy ε_*n***k**_. The integral in Eq. 3 is taken over the unit cell with real-space area Ω. Correspondingly, the displacement field due to the free charges is given by **D**_**G**_(**q**) = $\sum_{G^{\prime }}\epsilon_{{GG}^{\prime }}$(**q**, ω)$E_{G^{\prime }}$(**q**), where $E_{G^{\prime }}$(**q**) are the Fourier components of the total electric field in the crystal. Plasmon, a sustained electric-field oscillation in the absence of free charges [**D**_**G**_(**q**) = 0] is given by the solution of a zero eigenvalue problem det ε_**GG**′_ = 0. The relevant eigenvectors correspond to the real-space pattern of charge oscillations. Note that because of the finite size of the unit cell compared to the electromagnetic wave vector, the fields in free space do not map simply (*42*, *43*) to the field in the crystal **E**_**G**_(**q**) due to the additional dependence on reciprocal lattice vectors (see the Supplementary Materials for further discussion). In a typical tight-binding model, where the cell-periodic part of the Bloch function becomes position independent [*u*_*n***k**_(**r**) = *u*_*n***k**_], the overlap of Eq. 3 vanishes for any **G** ≠ 0, making the notion of local field effects irrelevant (only ε_00_(**q**, ω) is nonzero in Eq. 1). This property is manifestly not true in continuum models of moiré materials where the cell-periodic part of the Bloch functions is position dependent, as we detail below.

### Model

To describe our platform of choice, the WSe_2_/WS_2_ heterobilayers with zero twist angle, we use the Bistritzer-MacDonald–type continuum model (*40*). One of the key benefits of the heterobilayer setup is that, because of the lattice mismatch, the moiré pattern is present even without any twisting angle between layers. This makes the system robust to moiré potential disorder as the moiré lattice constant is not as sensitive to small variations in layer placement. The valence moiré bands of this platform are composed of WSe_2_ hole pockets centered around K and K′ points of the BZ. Owing to the spin-valley locking of TMDs (*52*), the K/K′ valley degree of freedom can be identified with the electron spin, and the system is thus doubly degenerate. Within each valley, the Hamiltonian *H*_0_ can be approximated by a parabolic band with periodic moiré potential *V_M_*(**r**) resulting from the insulating layer of WS_2_ imposed on top of it (*49*, *53*)

$$H_{0}=-\frac{\hbar^{2}k^{2}}{2m^{*}}+V_{M}(r)$$ (4)

where m* = 0.472 *m_e_* is the effective mass of the hole pocket of WSe_2_. The moiré potential itself can be expressed in terms of its Fourier components corresponding to the first shell of reciprocal lattice vectors **b***_i_*, with $b_{1}=2\pi /a_{M}[2/\sqrt{3},0]$, and the remaining five vectors obtained by π/3 rotations. Here *a_M_* = *a*/δ ≈ 8.2 nm is the moiré lattice constant where *a* = 0.328 nm and δ = 4% are the lattice constant of WSe_2_ and lattice mismatch between WSe_2_/WS_2_, respectively (*49*, *53*). The moiré potential can thus be expanded as

$$V_{M}(r)=\sum_{j}V_{b_{j}}e^{ib_{j}\cdot r}$$ (5)

where *V*_**b**_1__ = *V*_0_*e*^*i*ψ^, with *V*_0_ = 15 meV and ψ = 45° for WSe_2_/WS_2_ (*53*), and the remaining coefficients can be obtained from symmetry as *V*_**b**_ = *V*_*R*(2π/3,**b**)_ and $V_{b}=V_{-b}^{*}$ [here, *R*(θ, **b**) denotes counterclockwise rotation of vector **b** by θ].

The moiré potential breaks down the parabolic band from Eq. 4 into a series of mini-bands defined within the moiré BZ, as shown in fig. S1. The charge neutrality point of the entire structure lies within the bulk gap of WSe_2_ and WS_2_; thus, the topmost doubly degenerate valence moiré band (with the narrow band width of below 10 meV) corresponds to the filling −2 ≤ ν ≤ 0 as defined in the experiments (*54*). To study the fillings 0 ≤ ν ≤ 2, one can also use a similar model, which, with an appropriate adjustment to the effective mass, describes moiré conduction bands formed by WS_2_ electron pockets. In what follows when referring to a filling of ν, we are considering a hole filling of valence bands −ν (i.e., the top valence band has 2 − ν electrons) or an electron filling ν of the bottom conduction band (i.e., the band has ν electrons in it). We also refer to these top valence or bottom conduction bands as the moiré flat band. Because the model of Eq. 4 describes the low energy theory of the entire moiré TMD bilayer and not just the WSe_2_, it is therefore sufficient at the energy scales and frequencies of interest to describe the system with a single dynamical dielectric function as opposed to a separate dielectric function for each TMD layer.

To understand the origins of the correlated phases present in the TMD moiré structures, the flat band of the model can be considered within the framework of a Hubbard model on a triangular lattice (*48*–*51*). The correlated phases can then be obtained using the Hartree-Fock mean-field treatment (*48*, *50*, *55*), and, depending on the strength of interactions and filling of the bands, the ground state can, for example, be ferromagnetic (FM) or antiferromagnetic (AFM) with various patterns of in-plane or out-of-plane magnetic moments. In particular, we focus on the two key characteristics of the ground states that, as we will see, are relevant for the qualitative properties of the dielectric response: the enlargement of the unit cell due to the formation of a correlated phase, and what spin structure the ground state has (cf. Fig. 1A). Specifically, building on the observation that the Hartree-Fock ground states have a generalized Wigner crystal character (*48*, *49*) with charge mainly localized around selected moiré potential minima, we use a Hartree-like electrostatic potential coming from the charges localized in the moiré potential minima and effective magnetic fields that are consistent with the orientation of the magnetic moments as shown in the self-consistent calculations. This approach, together with the enlargement of the unit cell due to the formation of the Wigner crystal and using experimentally obtained estimates for the magnitudes of the correlated gaps (*54*), enables us to give an overview of the qualitative features observed in the dynamic dielectric response of the system that can help us to identify the nature of the correlated ground state. We caution in passing that Hartree-Fock techniques are an approximate scheme, and, thus, if a more exact method is used, then different candidate ground states may become relevant. Such analysis would not, however, influence the conclusions of our work as its goal is to demonstrate that different ground states may be distinguished using plasmons, rather than to provide a theoretical prediction of the actual experimentally realized ground state. We thus use the mean-field calculation as a guide to what are the expected correlated orders that we can use as a starting point for the plasmon spectrum calculation.

Expressing the Hamiltonian *H*_0_ in terms of reciprocal lattice vectors of the original moiré lattice **G**_0_, we have

$$H_{0}^{G_{0}G_{0}^{\prime }}(k)=-\frac{{|k+G_{0}|}^{2}}{2m^{*}}\delta_{G_{0}G_{0}^{\prime }}+\sum_{i}V_{b_{i}}\delta_{G_{0}-G_{0}^{\prime },b_{i}}$$ (6)

When a generalized Wigner crystal state appears, it is pinned to a fraction of the sites of the underlying moiré lattice, with the overall periodicity of that lattice different for each correlated ground state (*48*, *50*, *55*). This translates to a different set of reciprocal lattice vectors **G**, which are shorter than moiré reciprocal lattice (i.e., moiré BZ becomes folded). In terms of the vectors **G**, the Hartree (charge) potential takes the form

$$H_{C}^{GG^{\prime }}(k)=V_{C}\sum_{\tau }e^{-i(G-G^{\prime })\cdot \tau }V_{G-G^{\prime }}$$ (7)

where *V_C_* is a fitting parameter chosen to reproduce the experimental gap at a given filling as detailed in the concrete examples below, and τ are the crystal basis vectors within the enlarged unit cell that specify the centers of charge distribution for a given filling. Similarly, we take the effective potential that enforces the spin texture predicted by Hartree-Fock calculations (*48*, *55*) as given by

$$H_{M}^{GG^{\prime }}(k)=V_{B}\sum_{\tau }e^{-i(G-G^{\prime })\cdot \tau }B(G-G^{\prime },\tau )\;\cdot \sigma$$ (8)

Here, *V_B_* is also a fitting parameter, **B**(**G** − $G^{\prime }$, τ) is a vector that determines the orientation of the magnetic field at a given crystal basis site τ, and σ is a vector of Pauli matrices that describes the spin degree of freedom. More details of the model, together with the parameters used in the calculations, are presented in Materials and Methods.

Because of the folding of the moiré BZ, new electron bands form that originate from the moiré flat band. Once the effective charge (Eq. 7) and spin (Eq.8) potentials are included in the Hamiltonian, gaps open between these folded bands. We, however, highlight that, in each case, the model is only valid when the chemical potential is placed within the gap that corresponds to the filling fraction consistent with the ground state used. With the phenomenological models for the correlated states described above, we can now calculate the dielectric function matrix with the local field effects included. We perform the calculations for several distinct filling fractions ν = 1, 2/3, 1/2, 1/3 for different candidate ground states, which correspond to the most prominent generalized Wigner crystals as observed in the experiments (*54*).

### Plasmon folding

The central result of our work, the appearance of multiple plasmon resonances stemming from the local field effects and their strong dependence on the type of correlated order, is shown in Fig. 1 (B and C). This behavior can be understood by studying the structure of the dielectric function ε_**GG**′_(**q**, ω), which, in general, has two types of entries: diagonal (**G** = $G^{\prime }$) and off-diagonal (**G** ≠ $G^{\prime }$). The diagonal entries when expressed using the polarization function Π(**q**, ω) have the characteristic RPA structure, 1 − *V*_**q**_Π(**q**, ω) ≡ 1 − *T*_**GG**_(**q**, ω), each, in principle, yielding a collective excitation solution ω*_n_*(**q**) when taken separately through 1 = *T*_**GG**_[**q**, ω*_n_*(**q**)] where the index *n* labels each solution. The off-diagonal entries for frequencies outside of the particle-hole continua are of a fixed sign. As such, the diagonal entries give rise to the multiple folded plasmon branches, and the off-diagonal entries of the dielectric function matrix open gaps between them.

To see this explicitly, let us focus on the behavior of ε_**GG**′_(**q**, ω) near the BZ edge at **q***_M_* = −**G**_**1**_/2 (chosen to be the *M* point of the hexagonal BZ) for the case of ν = 2/3 filling shown in Fig. 1C. In particular, we focus on a ground state that is a pure Wigner crystal (*V_C_* ≠ 0) without a spin structure (*V*_B_ = 0). Near **q***_M_*, only the matrix entries corresponding to momenta **G** = 0, **G**_**1**_ contribute to the leading order in the ε_**GG**′_(**q**, ω) due to the Coulomb prefactor 1/∣**q** + **G**∣. The resulting dielectric function matrix has the structure

$$\varepsilon_{{GG}^{\prime }}(q,\omega )=\left[\begin{array}{cc} 1-T_{00}(q,\omega ) & -T_{0G_{1}}(q,\omega )\\ -T_{G_{1}0}(q,\omega ) & 1-T_{G_{1}G_{1}}(q,\omega ) \end{array}\right]$$ (9)

For simplicity of the argument in evaluating the function *T*_**GG**′_(**q**, ω) of Eq. 1, we can focus on the electron states closest in energy, i.e., bands separated by the correlated gap Δ. To leading order in momentum **q**, we can approximate the energy difference ∣ε_*n***k**_ − ε_*m***k**+**q**_∣ ≈ Δ yielding

$$\varepsilon_{{GG}^{\prime }}(q,\omega )\approx \left[\begin{array}{cc} 1-\frac{A(q)}{\omega^{2}-{\text{Δ}}^{2}} & -\frac{C(q)}{\omega^{2}-{\text{Δ}}^{2}}\\ -\frac{D(q)}{\omega^{2}-{\text{Δ}}^{2}} & 1-\frac{B(q)}{\omega^{2}-{\text{Δ}}^{2}} \end{array}\right]$$ (10)

In arriving at this expression, we used time-reversal symmetry of the system that requires ω → −ω [see the Supplementary Materials for a careful derivation, and see also (*56*)]. The resulting plasmon dispersion [obtained from det ε_**GG**′_(**q**, ω) = 0; see Fig. 1C] is given by

$$\omega_{\pm }^{2}(q)={\text{Δ}}^{2}+\frac{1}{2}\left[A+B\pm \sqrt{(A-B{)}^{2}+4CD}\right]$$ (11)

where we suppress the dependence on momentum in *A*, *B*, *C*, and *D* for clarity. We see that, in the absence of the off-diagonal entries (*C* = *D* = 0), there are two plasmon solutions, $\omega_{-}^{2}={\text{Δ}}^{2}+A(q)$ and $\omega_{+}^{2}={\text{Δ}}^{2}+B(q)$ (orange lines in Fig. 1B). Off-diagonal entries hybridize the two solutions opening the gap Δ_P_ ≡ ω_+_(**q***_M_*) − ω_−_(**q***_M_*) in the plasmon spectrum at the boundary. The other plasmon resonances seen in Fig. 1B originate similarly from the diagonal entries of ε_**GG**′_(**q**, ω) (see movie S1); however, the energy ordering in which the individual resonances appear once off-diagonal entries are introduced is nongeneric. We discuss both of these points in the following paragraphs.

### Plasmon folding as a signature of correlated states

The appearance of multiple plasmon branches is a robust feature expected simply due to the folding of the moiré BZ owing to the correlated order enlarging the unit cell (Fig. 1A). To see this explicitly, consider the bare Hamiltonian of Eq. 4 (*V_C_* = 0, *V_B_* = 0) but with an artificial enlargement of a unit cell to that of a unit cell of the ν = 2/3 Wigner crystal. The resulting plasmon resonance (Fig. 1C) is folded at the new BZ edge (cf. Fig. 1B), with a gap opening up in the plasmon dispersion when an actual correlated order appears (see movie S2), in addition to the opening of a gap Δ in the band structure that gaps out the conventional intraband plasmon as **q** → 0.

We pause here to comment on the key claim of our paper, which attributes the appearance of the plasmon folding solely to the correlated order. The dielectric function matrix is a well-studied concept, both in first-principle calculations and early works on properties of nearly free electron systems (*42*–*44*). Oliveira and Sturm (*47*) first predicted the appearance of the folded plasmon resonance, albeit for a narrow momentum window (greatly limited by damping) in a nearly free 3D electron gas. In the flat-band systems, because of the mismatch between the kinetic and Coulomb energy scales, plasmon dispersion rises above the particle-hole continuum (*57*–*61*), circumventing the problem encountered by Oliveira and Sturm (*47*). At first glance, therefore, a metallic moiré flat-band system should exhibit such folded plasmon resonances. This, however, does not occur due to the subtle interplay of interband and intraband contributions to the polarization functions, as we explain below.

When local field effects are introduced in a metallic moiré flat-band system (cf. Fig. 1D), the plasmon dispersion flattens and is pushed to higher energies near the BZ, but a new plasmon branch does not appear. Cea and Guinea (*62*) found similar behavior in the case of the twisted bilayer graphene continuum model, lacking any additional plasmon resonances. The origin of this behavior can be traced to the form of the dielectric response of the Eq. 9. Schematically, the **G**_1_**G**_1_ entry can be found by extending the 00 entry to the second BZ and then folding it back into the first BZ as depicted in Fig. 1B. Suppose the plasmon dispersion has a maximum away from the BZ boundary. In that case, the resulting folded branch (i.e., found only by keeping the diagonal entries of the dielectric matrix) will appear below the original plasmon branch. In turn, when the off-diagonal elements of the dielectric matrix are considered, the resulting gap between the original (now higher energy) branch and that of the folded (now lower energy) branch pushes the folded branch into the particle-hole continuum and the original branch upward in energy, flattening it near the BZ boundary as shown in Fig. 1D (see also movie S3). On the other hand, suppose the original plasmon branch has a maximum at the BZ edge, as it does in the schematic of Fig. 1B. In that case, the folded plasmon will appear in the BZ above the original plasmon branch. The off-diagonal element of the dielectric matrix will then separate the two plasmons that are not pushed into the particle-hole continuum.

A nontrivial question therefore arises: What controls the overall shape of the plasmon dispersion? Or, more precisely: Where does its maximum in dispersion lie in relation to the BZ edge? Because of the interplay of two types of entries in the polarization function, as discussed by Lewandowski and Levitov (*57*), those corresponding to transitions between the states separated by energies higher than the plasmon resonance (type I), and those corresponding to transitions between states separated by energies lower than the plasmon resonance (type II). The type II transitions give rise to the plasmon dispersion $\propto \sqrt{q}$, while the type I transitions act to renormalize the plasmon dispersion at large momenta comparable to the BZ scale. As a result of this interplay, in a generic metallic moiré flat-band system, a maximum in the plasmon dispersion will occur away from the BZ boundary. When a correlated order appears, the maximum of the plasmon dispersion is pushed toward the BZ zone interface, and the plasmon becomes flattened. This process, in conjunction with the reduction of the BZ size, gives rise to the folding of plasmons, which are flattened at large momenta and, hence, produce many weakly dispersing plasmon resonances. This rich interplay of type I and type II transitions with local field effects appears only in multiband continuum models and thus is missed by a preliminary tight-binding-based analysis as studied in (*63*), which predicts folding of a plasmon branch in any two-site model, e.g., both metallic twisted bilayer graphene and a Mott insulator would manifest folded plasmon branches.

### Plasmon spectrum of a generalized Wigner crystal

**Fig. 2.**
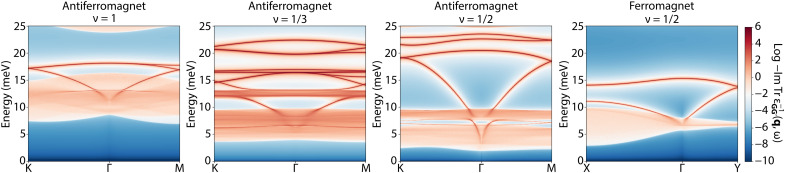
The trace of the electron loss function at ν = 1,1/3,1/2 hole filling fractions. In each case, multiple plasmon branches are present due to local field effects. The enlargement of the moiré lattice unit cell in the correlated state determines thenumber of the plasmon branches. The size of the interacting gap determines the lower bound of interband particle-hole continua. In the case of ν = 1/2, the two competing states are FM and AFM, and due to their different lattice periodicity, they have radically different plasmonic spectra.

We can now analyze the different dynamical responses for the various filling fractions and candidate ground states in more detail; see Fig. 2. Unlike the uncorrelated state, Fig. 1D, here, for any of the fractional fillings, we find several plasmon branches with the precise number set by the underlying ground state. More specifically, on the basis of the arguments of the previous section, if only the diagonal entries were considered, then we should see many plasmon branches ε_**GG**′_(**q**, ω). Here, however (despite considering more than 40 reciprocal lattice vectors in the presented results), we see substantially fewer resonances, i.e., the off-diagonal components of the dielectric function push other resonances into the particle-hole continua. Unexpectedly, we also find that the number of plasmon resonances (degenerate along some high-symmetry directions) is precisely equal to the enlargement of the unit cell compared to the original moiré BZ; i.e., for ν = 1, we expect 3; for ν = 1/3, we expect 9; for ν = 1/2 (AFM) we expect 4; and for ν = 1/2 (FM), we expect 2. We postulate this to be a key signature that enables the detection of the underlying lattice enlargement.

One can understand this parallel between the number of plasmon branches and the unit cell enlargement in the following way. In the absence of a correlated state, only one plasmon branch appears within the moiré BZ as shown in Fig. 1D, even when local field effects are taken into account. As explained above, this is because the plasmon branch arising from ε_00_ is sloped downward in the vicinity of the BZ boundary. Thus, when new plasmon branches appear with other diagonal elements of ε_**GG**′_ taken into account, they will be lower in energy than the original ε_00_ branch. As off-diagonal elements are included in the calculation, they will open gaps between various plasmon branches, pushing the original branch up and all the other branches down into the particle-hole continuum. As the moiré potential is an energy scale larger than the plasmon energies themselves, it buries all but one plasmon branch in the continuum. This is clearly visualized in movie S3, where the off-diagonal elements gradually increase, and the process of removing the other plasmon branches is evident. This one remaining plasmon is then the source of the multiple plasmon branches in the Wigner crystal state. A similar mechanism of pushing plasmons into particle hole-continua can also be observed in the case when we switch on the off-diagonal terms in the case with ν = 2/3 Wigner crystal (see movie S1), but because the energy scale of the Wigner crystal is smaller than that of the moiré potential and, in consequence, smaller than the plasmon energy, the inclusion of the Hartree-like potential leads to opening of the gaps in plasmon spectrum but does not lead to the disappearance of the plasmon branches. As a result, because only one (the original moiré) plasmon becomes folded as many times as the original moiré BZ is folded, we expect the number of plasmon branches to be set by the enlargement of the BZ.

The details of the plasmon dispersion behavior and the overall dynamical response (specifically, the particle-hole continuum) can yield further insights into the nature of the underlying ground state. The interband continuum reveals the presence and magnitude of the correlated gaps as it extends down to the lowest energies in the metallic case. Similarly, the plasmon dispersion in the metallic case stretches down to zero frequency as **q** → 0. In contrast, when interactions open the gap, the plasmon also rises to higher energies, even for the smallest momenta, as discussed in the context of Fig. 1 (B and C). As the moiré materials allow for continuous tuning of the filling fraction, this opening of the gap could be directly observed when the plasmon spectrum is measured.

Our method can also help distinguish between different types of correlated ground states that can appear at the same filling. For example, at ν = 1/2, the generalized Wigner crystal forms stripes, separated by two moiré lattice constants (*48*). However, such stripes may have either FM or AFM chain configurations. While in the FM state, the lattice period becomes doubled only in one direction, the AFM state has a doubling of both lattice vectors. This corresponds to two and four times smaller BZs for FM and AFM, respectively. This, as shown in Fig. 2, has marked consequences for the dielectric response. Not only the plasmon energies are entirely different, but even the number of plasmon branches changes between the two states, as mentioned previously. This enables distinguishing the type of ground state present in the system.

The behavior of the plasmon dispersion near the folded BZ edge yields further insights into the nature of the underlying order. In Fig. 2, we see that the plasmon dispersion at the *M* point does not have a gap. Within the 2 × 2 effective dielectric function model of Eq. 9, a gap in the plasmon spectrum will not open if the off-diagonal entry of the dielectric function matrix vanishes. This is the case for Fig. 2 (ν = 1/2 AFM) at the high-symmetry point *M*, which is in the direction of the reciprocal vector **G**_1_ yielding ε_0**G**_1__(ω, **q** = −**G**_1_/2) = 0 and ε_**G**_1_0_(ω, **q** = −**G**_1_/2) = 0. This is in contrast to an inequivalent high-symmetry point *M*′ in the direction **G**_2_ where a gap does open (see fig. S3) as the corresponding off-diagonal entries do not vanish. This vanishing of the off-diagonal entry for some **G** value is not a consequence of vanishing state overlaps of Eq. 3 for all BZ **k** points (as other entries of the ε_**GG**′_ feature them as well), but instead such entry vanishes only after summation over the BZ as a consequence of symmetry of the ν = 1/2 AFM state. More precisely, we attribute this to the striped nature of the ν = 1/2 state where period doubling occurs only along one direction, breaking the *C*_3_ symmetry present for other generalized Wigner crystals considered. The extent to which the vanishing of the off-diagonal entries and hence plasmon gap closing is a general feature of the ν = 1/2 correlated state remains to be assessed based on different microscopic models.

**Fig. 3.**
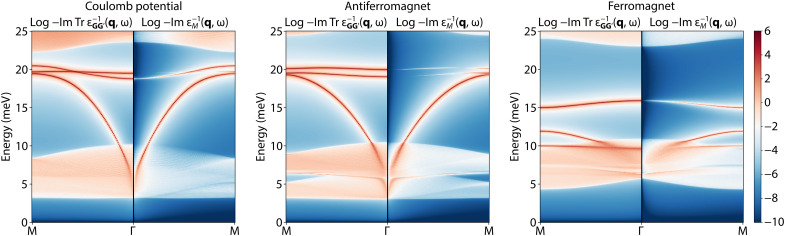
The comparison between the trace of the energy loss function and the inverse macroscopic dielectric function ε*_M_*(q,ω). Here, each panel corresponds to a different ground state at filling ν = 2/3. Although, in each case, the unit cell of the correlated state is the same (triple the size of the moiré unit cell), the plasmon spectra and particle-hole continua are distinct. While the trace of the loss functions shows all of the possible collective excitations of the system, the macroscopic dielectric function shows the optical absorption spectrum.

However, even if the unit cell is the same for two magnetic structures at the same filling, it is still possible to differentiate them on the basis of the plasmon spectrum. This is demonstrated in Fig. 3, where we show the results at ν = 2/3 for three different correlated states. They each have the same effective unit cells but have either purely electrostatic potential, an AFM state, or a FM state, and in each case, the correlated gap is set to 2.9 meV. Apart from the differences in plasmon dispersion, there is another notable contrast between the FM and AFM states: Because of the spin-polarization in the FM state, some excitation processes are forbidden due to the vanishing matrix elements of band overlap. This leads to an effective gap of 3.9 meV as determined by the boundaries of the particle-hole continuum. Such a gap would thus be different from the one observed in STM or activated transport experiments, where the matrix elements do not play a role. This adds an additional feature through which the dielectric response can identify these ground states.

### Experimental detection

Throughout the manuscript, we plotted the imaginary part of the trace of the inverse dielectric function, $\mathrm{Tr}\;\varepsilon_{GG^{\prime }}^{-1}(q,\omega )\propto 1/\det\;\varepsilon_{{GG}^{\prime }}(q,\omega )$, which carries information about all plasmon resonances [detε_**GG**′_(**q**, ω) = 0]. This form was chosen because of the general focus of the manuscript on the plasmon folding due to the formation of the correlated order. It is, however, an experimentally relevant question how and if all of those plasmon resonances can be excited in a given experimental setup. In particular, different plasmon launching schemes (*35*, *36*, *64*) could couple to the dielectric tensor (Eq. 1) differently. A detailed answer to this question depends on the specific experimental method used, as different techniques may excite different patterns of charge oscillations (e.g., dipole). Here, we focus on the long-wavelength limit, which corresponds to the conventional optical probes (**q** → 0), where the relevant quantity is the loss function defined (*65*) as $\log-\varepsilon_{M}^{-1}(q,\omega )$. Here, ε*_M_*(**q**, ω) is the macroscopic dielectric function (*42*–*44*)

$$\frac{1}{\varepsilon_{M}(q,\omega )}={\left[\varepsilon_{{GG}^{\prime }}^{-1}(q,\omega )\right]}_{00}$$ (12)

that is the **G** = $G^{\prime }$ = 0 entry of the inverse of the dielectric function from Eq. 1. This loss function describes the probability of exciting plane wave longitudinal charge oscillations. In Fig. 3, we compare the spectrum of the plasmon excitations to the plasmons that can be excited by conventional optical probes. We see that while some of the folded plasmons remain in the optical excitation spectrum with varying intensity, some of the resonances are not active and likely require experimental probes that can excite charge oscillations where individual Fourier wave vectors *E*_**G**_ have an internal structure (are not plane wave-like). We leave detailed consideration of these effects to future work.

## DISCUSSION

In summary, we have shown that the dynamical dielectric response, particularly the plasmon dispersion, can be a valuable tool in identifying correlated phases of moiré materials. The advent of tunable on-chip THz radiation sources presents an opportunity to clarify the nature of the rich phase diagram of correlated states in flat-band materials. Moreover, the results that we show here only constitute the first step in investigating the phenomenon of plasmon folding. One possible future direction is the investigation of real space patterns of charge density oscillations, which could be observed using scanning near-field optical microscopy (*35*, *36*, *66*, *67*).

As in our analysis we used the RPA approximation (Eq. 2), a natural question is to what extent our conclusions are valid in the strongly interacting (low-density) regime of the moiré materials. Crucially, the plasmon folding that we predict is a consequence of the dielectric function having a matrix structure owing to the inclusion of local field effects rather than the result of the RPA approximation. This matrix structure of the dielectric function will naturally give rise to the folding of a plasmon resonance when the correlated order appears, provided that such resonance exists in the original metal before the impact of the correlated ground state is considered. As argued by Lewandowski and Levitov (*57*) on general grounds based on Ward identities (namely, gauge invariance demanding that spatially uniform external potential does not perturb density), one expects a plasmon to exist with a $\lambda \sqrt{q}$ dispersion (with higher momentum correction becoming relevant at larger *q*) with the prefactor λ dependent on the details of the polarization function. As such, this plasmon will become folded due to the local field effects structure, making our predictions robust. We caution, however, that the quantitative features of the plasmon dispersion likely depend on the extent to which the RPA approximation captures the quantitative aspects of the exact density-density pair correlator. It would, therefore, be interesting to investigate beyond-RPA results in future work.

While the folding of the plasmon spectrum is a robust phenomenon stemming from the physics of BZ folding, the overall dispersion is sensitive to the details of the correlated order. In particular, in our analysis, we assume a simplified framework wherein the structure of the correlated order has no dependence on BZ momentum **q**. This is an oversimplification of the analysis, which a calculation that involves Hartree-Fock treatment could remedy. Such a calculation could also treat RPA screening with Hartree-Fock self-consistently (*68*, *69*) to conserve the particle number and imbue the plasmons with an additional momentum and/or spin dependence. In particular, moiré systems can in principle exhibit other collective modes (e.g., spin waves), which can similarly be obtained through the framework of RPA analysis. Thus, consideration of “local field effects” in the context of such excitations may prove a fruitful research direction to follow, potentially yielding alternative probes into the microscopic nature of the correlated states in the moiré materials.

## MATERIALS AND METHODS

### Ground states and parameters

This section provides additional details of the models used to calculate the energy loss functions for various filling fractions. In all the cases, the starting point is the moiré lattice with unit vectors given by

$$a_{1}=a_{M}(\sqrt{3}/2,\;-1/2),\;a_{2}=a_{M}(0,\;-1)$$ (13)

where *a_M_* = 8.2 nm is the moiré lattice constant. This triangular lattice then has a corresponding reciprocal lattice defined by the vectors

$$b_{1}=2\pi /a_{M}(2/\sqrt{3},\;0),\;b_{2}=2\pi /a_{M}(-1/\sqrt{3},\;-1)$$ (14)

The band structure of the noninteracting model is given in fig. S1. We then impose various generalized Wigner crystal states on top of this lattice, following the mean-field Hartree-Fock solutions of the triangular lattice Hubbard model from (*48*, *55*). The particular structures for each filling are presented in fig. S2, and the parameters used in the calculations are listed in table S1. In each case, the number of **G** vectors of the reciprocal lattice used in the calculation of ε_**GG**′_ was determined by imposing a cutoff of 3.55 **b**_*W*1_, corresponding to including above 40 shortest vectors.

The fitting parameters *V_C_* and *V_B_* are, in each case, chosen to give electronic bands with a bandgap estimated from the critical temperatures observed for correlated phases in (*54*). The Hartree-like potential is expressed in terms of the Fourier components of Coulomb interaction for **G** ≠ $G^{\prime }$ (with *V*_**G**−**G**′_ = 0 for **G** = $G^{\prime }$) and normalized by its value for **b**_*W*2_, one of the reciprocal lattice vectors of the generalized Wigner crystal lattice

$$V_{G-G^{\prime }}=\frac{2\pi e^{2}}{|G-G^{\prime }|}/\frac{2\pi e^{2}}{|b_{W2}|}$$ (15)

We choose the effective magnetic potential to be described as a combination of Gaussian functions in real space. Each is centered around the appropriate moiré lattice site given by the basis of Wigner crystal τ. It can be thus expressed in terms of the Fourier components as

$$B(G-G^{\prime },\tau )=\exp\left(-\frac{{|G-G^{\prime }|}^{2}}{2{|b_{W2}|}^{2}}\right)\hat{B}(\tau )$$ (16)

where $\hat{B}(\tau )$ is a unit vector in the direction given by the arrows shown in fig. S2, with purple arrows pointing in the *xy* plane, while red and blue arrows point along the *z* axis, perpendicular to the Wigner crystal.

In each calculation, the temperature is set to be *k*_B_*T* = 5 × 10^−5^ eV, and the chemical potentials are placed around the center of the gaps that result in the filling under study. We also consider the insulating material of the gate below the sample by setting the dielectric constant κ = 3. Changing this value does not affect the results qualitatively; the effect of increasing κ is the decrease of plasmon energies, with the dispersion becoming flatter. The summations over momenta within the BZ are performed over Mokhorst-Pack meshes (*70*) with 51 × 51 points in the Wigner crystal reciprocal unit cell, except for the unfolded case, where the mesh is 101 × 101.

## References

[1] L. Balents, C. R. Dean, D. K. Efetov, A. F. Young, Superconductivity and strong correlations in moiré flat bands. Nat. Phys. 16, 725–733 (2020).

[2] E. Y. Andrei, A. H. MacDonald, Graphene bilayers with a twist. Nat. Mater. 19, 1265–1275 (2020). 33208935 10.1038/s41563-020-00840-0

[3] K. F. Mak, J. Shan, Semiconductor moiré materials. Nat. Nanotechnol. 17, 686–695 (2022). 35836003 10.1038/s41565-022-01165-6

[4] Y. Cao, V. Fatemi, A. Demir, S. Fang, S. L. Tomarken, J. Y. Luo, J. D. Sanchez-Yamagishi, K. Watanabe, T. Taniguchi, E. Kaxiras, R. C. Ashoori, P. Jarillo-Herrero, Correlated insulator behaviour at half-filling in magic-angle graphene superlattices. Nature 556, 80–84 (2018). 29512654 10.1038/nature26154

[5] Y. Cao, V. Fatemi, S. Fang, K. Watanabe, T. Taniguchi, E. Kaxiras, P. Jarillo-Herrero, Unconventional superconductivity in magic-angle graphene superlattices. Nature 556, 43–50 (2018). 29512651 10.1038/nature26160

[6] X. Lu, P. Stepanov, W. Yang, M. Xie, M. A. Aamir, I. Das, C. Urgell, K. Watanabe, T. Taniguchi, G. Zhang, A. Bachtold, A. H. MacDonald, D. K. Efetov, Superconductors, orbital magnets and correlated states in magic-angle bilayer graphene. Nature 574, 653–657 (2019). 31666722 10.1038/s41586-019-1695-0

[7] M. Yankowitz, S. Chen, H. Polshyn, Y. Zhang, K. Watanabe, T. Taniguchi, D. Graf, A. F. Young, C. R. Dean, Tuning superconductivity in twisted bilayer graphene. Science 363, 1059–1064 (2019). 30679385 10.1126/science.aav1910

[8] G. Chen, L. Jiang, S. Wu, B. Lyu, H. Li, B. L. Chittari, K. Watanabe, T. Taniguchi, Z. Shi, J. Jung, Y. Zhang, F. Wang, Evidence of a gate-tunable Mott insulator in a trilayer graphene moiré superlattice. Nat. Phys. 15, 237–241 (2019).

[9] G. Chen, A. L. Sharpe, P. Gallagher, I. T. Rosen, E. J. Fox, L. Jiang, B. Lyu, H. Li, K. Watanabe, T. Taniguchi, J. Jung, Z. Shi, D. Goldhaber-Gordon, Y. Zhang, F. Wang, Signatures of tunable superconductivity in a trilayer graphene moiré superlattice. Nature 572, 215–219 (2019). 31316203 10.1038/s41586-019-1393-y

[10] A. Ghiotto, E.-M. Shih, G. S. S. G. Pereira, D. A. Rhodes, B. Kim, J. Zang, A. J. Millis, K. Watanabe, T. Taniguchi, J. C. Hone, L. Wang, C. R. Dean, A. N. Pasupathy, Quantum criticality in twisted transition metal dichalcogenides. Nature 597, 345–349 (2021). 34526705 10.1038/s41586-021-03815-6

[11] T. Li, S. Jiang, L. Li, Y. Zhang, K. Kang, J. Zhu, K. Watanabe, T. Taniguchi, D. Chowdhury, L. Fu, J. Shan, K. F. Mak, Continuous Mott transition in semiconductor moiré superlattices. Nature 597, 350–354 (2021). 34526709 10.1038/s41586-021-03853-0

[12] L. Wang, E.-M. Shih, A. Ghiotto, L. Xian, D. A. Rhodes, C. Tan, M. Claassen, D. M. Kennes, Y. Bai, B. Kim, K. Watanabe, T. Taniguchi, X. Zhu, J. Hone, A. Rubio, A. N. Pasupathy, C. R. Dean, Correlated electronic phases in twisted bilayer transition metal dichalcogenides. Nat. Mater. 19, 861–866 (2020). 32572205 10.1038/s41563-020-0708-6

[13] Y. Tang, L. Li, T. Li, Y. Xu, S. Liu, K. Barmak, K. Watanabe, T. Taniguchi, A. H. MacDonald, J. Shan, K. F. Mak, Simulation of Hubbard model physics in WSe_2_/WS_2_ moiré superlattices. Nature 579, 353–358 (2020). 32188950 10.1038/s41586-020-2085-3

[14] E. C. Regan, D. Wang, C. Jin, M. I. Bakti Utama, B. Gao, X. Wei, S. Zhao, W. Zhao, Z. Zhang, K. Yumigeta, M. Blei, J. D. Carlström, K. Watanabe, T. Taniguchi, S. Tongay, M. Crommie, A. Zettl, F. Wang, Mott and generalized Wigner crystal states in WSe_2_/WS_2_ moiré superlattices. Nature 579, 359–363 (2020). 32188951 10.1038/s41586-020-2092-4

[15] C. Jin, E. C. Regan, A. Yan, M. Iqbal Bakti Utama, D. Wang, S. Zhao, Y. Qin, S. Yang, Z. Zheng, S. Shi, K. Watanabe, T. Taniguchi, S. Tongay, A. Zettl, F. Wang, Observation of moiré excitons in WSe_2_/WS_2_ heterostructure superlattices. Nature 567, 76–80 (2019). 30804525 10.1038/s41586-019-0976-y

[16] K. L. Seyler, P. Rivera, H. Yu, N. P. Wilson, E. L. Ray, D. G. Mandrus, J. Yan, W. Yao, X. Xu, Signatures of moiré-trapped valley excitons in MoSe_2_/WSe_2_ heterobilayers. Nature 567, 66–70 (2019). 30804526 10.1038/s41586-019-0957-1

[17] K. Tran, G. Moody, F. Wu, X. Lu, J. Choi, K. Kim, A. Rai, D. A. Sanchez, J. Quan, A. Singh, J. Embley, A. Zepeda, M. Campbell, T. Autry, T. Taniguchi, K. Watanabe, N. Lu, S. K. Banerjee, K. L. Silverman, S. Kim, E. Tutuc, L. Yang, A. H. MacDonald, X. Li, Evidence for moiré excitons in van der Waals heterostructures. Nature 567, 71–75 (2019). 30804527 10.1038/s41586-019-0975-z PMC11493145

[18] E. M. Alexeev, D. A. Ruiz-Tijerina, M. Danovich, M. J. Hamer, D. J. Terry, P. K. Nayak, S. Ahn, S. Pak, J. Lee, J. I. Sohn, M. R. Molas, M. Koperski, K. Watanabe, T. Taniguchi, K. S. Novoselov, R. V. Gorbachev, H. S. Shin, V. I. Fal’ko, A. I. Tartakovskii, Resonantly hybridized excitons in moiré superlattices in van der Waals heterostructures. Nature 567, 81–86 (2019). 30842637 10.1038/s41586-019-0986-9

[19] H. Li, S. Li, E. C. Regan, D. Wang, W. Zhao, S. Kahn, K. Yumigeta, M. Blei, T. Taniguchi, K. Watanabe, S. Tongay, A. Zettl, M. F. Crommie, F. Wang, Imaging two-dimensional generalized Wigner crystals. Nature 597, 650–654 (2021). 34588665 10.1038/s41586-021-03874-9

[20] C. Rubio-Verdú, S. Turkel, Y. Song, L. Klebl, R. Samajdar, M. S. Scheurer, J. W. F. Venderbos, K. Watanabe, T. Taniguchi, H. Ochoa, L. Xian, D. M. Kennes, R. M. Fernandes, Á. Rubio, A. N. Pasupathy, Moiré nematic phase in twisted double bilayer graphene. Nat. Phys. 18, 196–202 (2022).

[21] Y. Cao, D. Rodan-Legrain, J. M. Park, N. F. Q. Yuan, K. Watanabe, T. Taniguchi, R. M. Fernandes, L. Fu, P. Jarillo-Herrero, Nematicity and competing orders in superconducting magic-angle graphene. Science 372, 264–271 (2021). 33859029 10.1126/science.abc2836

[22] T. Ideue, Y. Iwasa, Symmetry breaking and nonlinear electric transport in van der Waals nanostructures. Annu. Rev. Condens. Matter Phys. 12, 201–223 (2021).

[23] Y. Jiang, X. Lai, K. Watanabe, T. Taniguchi, K. Haule, J. Mao, E. Y. Andrei, Charge order and broken rotational symmetry in magic-angle twisted bilayer graphene. Nature 573, 91–95 (2019). 31365921 10.1038/s41586-019-1460-4

[24] A. Kerelsky, L. J. McGilly, D. M. Kennes, L. Xian, M. Yankowitz, S. Chen, K. Watanabe, T. Taniguchi, J. Hone, C. Dean, A. Rubio, A. N. Pasupathy, Maximized electron interactions at the magic angle in twisted bilayer graphene. Nature 572, 95–100 (2019). 31367030 10.1038/s41586-019-1431-9

[25] Y. Choi, J. Kemmer, Y. Peng, A. Thomson, H. Arora, R. Polski, Y. Zhang, H. Ren, J. Alicea, G. Refael, F. von Oppen, K. Watanabe, T. Taniguchi, S. Nadj-Perge, Electronic correlations in twisted bilayer graphene near the magic angle. Nat. Phys. 15, 1174–1180 (2019).

[26] D. Călugăru, N. Regnault, M. Oh, K. P. Nuckolls, D. Wong, R. L. Lee, A. Yazdani, O. Vafek, B. A. Bernevig, Spectroscopy of twisted bilayer graphene correlated insulators. Phys. Rev. Lett. 129, 117602 (2022). 36154402 10.1103/PhysRevLett.129.117602

[27] J. P. Hong, T. Soejima, M. P. Zaletel, Detecting symmetry breaking in magic angle graphene using scanning tunneling microscopy. Phys. Rev. Lett. 129, 147001 (2022). 36240422 10.1103/PhysRevLett.129.147001

[28] F. Xie, J. Kang, B. A. Bernevig, O. Vafek, N. Regnault, Phase diagram of twisted bilayer graphene at filling factor ν = ±3. Phys. Rev. B. 107, 075156 (2023).

[29] N. J. Zhang, J.-X. Lin, Y. Wang, K. Watanabe, T. Taniguchi, L. Fu, J. I. A. Li, Diodic transport response and the loop current state in twisted trilayer graphene. arXiv:2209.12964 [Preprint] [cond-mat.mes-hall] (26 September 2022).

[30] N. J. Zhang, Y. Wang, K. Watanabe, T. Taniguchi, O. Vafek, J. I. A. Li, Electronic anisotropy in magic-angle twisted trilayer graphene. arXiv:2211.01352 [Preprint] [cond-mat.mes-hall] (2 November 2022).

[31] A. N. Grigorenko, M. Polini, K. S. Novoselov, Graphene plasmonics. Nat. Photon. 6, 749–758 (2012).

[32] M. S. Tame, K. R. McEnery, Ş. K. Özdemir, J. Lee, S. A. Maier, M. S. Kim, Quantum plasmonics. Nat. Phys. 9, 329–340 (2013).

[33] W. L. Barnes, A. Dereux, T. W. Ebbesen, Surface plasmon subwavelength optics. Nature 424, 824–830 (2003). 12917696 10.1038/nature01937

[34] G. X. Ni, A. S. McLeod, Z. Sun, L. Wang, L. Xiong, K. W. Post, S. S. Sunku, B.-Y. Jiang, J. Hone, C. R. Dean, M. M. Fogler, D. N. Basov, Fundamental limits to graphene plasmonics. Nature 557, 530–533 (2018). 29795255 10.1038/s41586-018-0136-9

[35] J. Chen, M. Badioli, P. Alonso-González, S. Thongrattanasiri, F. Huth, J. Osmond, M. Spasenović, A. Centeno, A. Pesquera, P. Godignon, A. Zurutuza Elorza, N. Camara, F. J. G. de Abajo, R. Hillenbrand, F. H. L. Koppens, Optical nano-imaging of gate-tunable graphene plasmons. Nature 487, 77–81 (2012). 22722861 10.1038/nature11254

[36] Z. Fei, A. S. Rodin, G. O. Andreev, W. Bao, A. S. McLeod, M. Wagner, L. M. Zhang, Z. Zhao, M. Thiemens, G. Dominguez, M. M. Fogler, A. H. C. Neto, C. N. Lau, F. Keilmann, D. N. Basov, Gate-tuning of graphene plasmons revealed by infrared nano-imaging. Nature 487, 82–85 (2012). 22722866 10.1038/nature11253

[37] W. Zhao, S. Zhao, H. Li, S. Wang, S. Wang, M. I. B. Utama, S. Kahn, Y. Jiang, X. Xiao, S. Yoo, K. Watanabe, T. Taniguchi, A. Zettl, F. Wang, Efficient Fizeau drag from Dirac electrons in monolayer graphene. Nature 594, 517–521 (2021). 34163053 10.1038/s41586-021-03574-4

[38] Y. Dong, L. Xiong, I. Y. Phinney, Z. Sun, R. Jing, A. S. McLeod, S. Zhang, S. Liu, F. L. Ruta, H. Gao, Z. Dong, R. Pan, J. H. Edgar, P. Jarillo-Herrero, L. S. Levitov, A. J. Millis, M. M. Fogler, D. A. Bandurin, D. N. Basov, Fizeau drag in graphene plasmonics. Nature 594, 513–516 (2021). 34163054 10.1038/s41586-021-03640-x

[39] J. M. B. Lopes dos Santos, N. M. R. Peres, A. H. Castro Neto, Graphene bilayer with a twist: Electronic structure. Phys. Rev. Lett. 99, 256802 (2007). 18233543 10.1103/PhysRevLett.99.256802

[40] R. Bistritzer, A. H. MacDonald, Moiré bands in twisted double-layer graphene. Proc. Natl. Acad. Sci. U.S.A. 108, 12233–12237 (2011). 21730173 10.1073/pnas.1108174108 PMC3145708

[41] S. Carr, D. Massatt, S. Fang, P. Cazeaux, M. Luskin, E. Kaxiras, Twistronics: Manipulating the electronic properties of two-dimensional layered structures through their twist angle. Phys. Rev. B 95, 075420 (2017).

[42] N. Wiser, Dielectric constant with local field effects included. Phys. Rev. 129, 62–69 (1963).

[43] S. L. Adler, Quantum theory of the dielectric constant in real solids. Phys. Rev. 126, 413–420 (1962).

[44] M. Gajdoš, K. Hummer, G. Kresse, J. Furthmüller, F. Bechstedt, Linear optical properties in the projector-augmented wave methodology. Phys. Rev. B 73, 045112 (2006).

[45] W. M. Saslow, G. F. Reiter, Plasmons and characteristic energy loss in periodic solids. Phys. Rev. B 7, 2995–3003 (1973).

[46] K. Sturm, L. E. Oliveira, High-frequency dielectric properties of covalent semiconductors within the nearly-free-electron approximation. I. The one-plasmon-band model. Phys. Rev. B 22, 6268–6282 (1980).

[47] L. E. Oliveira, K. Sturm, High-frequency dielectric properties of covalent semiconductors within the nearly-free-electron approximation. II. The two-plasmon-band model. Phys. Rev. B 22, 6283–6293 (1980).

[48] H. Pan, F. Wu, S. Das Sarma, Quantum phase diagram of a Moiré-Hubbard model. Phys. Rev. B 102, 201104 (2020).

[49] F. Wu, T. Lovorn, E. Tutuc, A. H. MacDonald, Hubbard model physics in transition metal dichalcogenide moiré bands. Phys. Rev. Letters 121, 026402 (2018). 10.1103/PhysRevLett.121.026402 30085734

[50] J. Zang, J. Wang, J. Cano, A. J. Millis, Hartree-fock study of the moiré hubbard model for twisted bilayer transition metal dichalcogenides. Phys. Rev. B 104, 075150 (2021).

[51] K. Lee, P. Sharma, O. Vafek, H. J. Changlani, Triangular lattice Hubbard model physics at intermediate temperatures. arXiv:2209.00664 [Preprint] [condmat.str-el] (1 September 2022).

[52] D. Xiao, G.-B. Liu, W. Feng, X. Xu, W. Yao, Coupled spin and valley physics in monolayers of MoS_2_ and other group-VI dichalcogenides. Phys. Rev. Lett. 108, 196802 (2012). 23003071 10.1103/PhysRevLett.108.196802

[53] Y. Zhang, N. F. Q. Yuan, L. Fu, Moiré quantum chemistry: Charge transfer in transition metal dichalcogenide superlattices. Phys. Rev. B 102, 201115 (2020).

[54] Y. Xu, S. Liu, D. A. Rhodes, K. Watanabe, T. Taniguchi, J. Hone, V. Elser, K. F. Mak, J. Shan, Correlated insulating states at fractional fillings of moiré superlattices. Nature 587, 214–218 (2020). 33177668 10.1038/s41586-020-2868-6

[55] H. Pan, F. Wu, S. Das Sarma, Band topology, Hubbard model, Heisenberg model, and Dzyaloshinskii-Moriya interaction in twisted bilayer WSe_2_. Phys. Rev. Res. 2, 033087 (2020).

[56] H. Ishizuka, A. Fahimniya, F. Guinea, L. Levitov, Purcell-like enhancement of electron–phonon interactions in long-period superlattices: Linear-temperature resistivity and cooling power. Nano Lett. 21, 7465–7471 (2021). 34515488 10.1021/acs.nanolett.1c00565

[57] C. Lewandowski, L. Levitov, Intrinsically undamped plasmon modes in narrow electron bands. Proc. Natl. Acad. Sci. U.S.A. 116, 20869–20874 (2019). 31562197 10.1073/pnas.1909069116 PMC6800336

[58] P. Novelli, I. Torre, F. H. L. Koppens, F. Taddei, M. Polini, Optical and plasmonic properties of twisted bilayer graphene: Impact of interlayer tunneling asymmetry and ground-state charge inhomogeneity. Phys. Rev. B 102, 125403 (2020).

[59] K. Khaliji, T. Stauber, T. Low, Plasmons and screening in finite-bandwidth two-dimensional electron gas. Phys. Rev. B 102, 125408 (2020).

[60] X. Kuang, Z. Zhan, S. Yuan, Collective excitations and flat-band plasmon in twisted bilayer graphene near the magic angle. Phys. Rev. B 103, 115431 (2021).

[61] M. Papaj, C. Lewandowski, Plasmonic nonreciprocity driven by band hybridization in moiré materials. Phys. Rev. Lett. 125, 066801 (2020). 32845684 10.1103/PhysRevLett.125.066801

[62] T. Cea, F. Guinea, Coulomb interaction, phonons, and superconductivity in twisted bilayer graphene. Proc. Natl. Acad. Sci. U.S.A. 118, e2107874118 (2021). 34362849 10.1073/pnas.2107874118 PMC8364166

[63] A. Fahimniya, C. Lewandowski, L. Levitov, Dipole-active collective excitations in moiré flat bands. arXiv:2011.02982 [Preprint] [cond-mat.mes-hall] (5 November 2020).

[64] D. A. Bandurin, D. Svintsov, I. Gayduchenko, S. G. Xu, A. Principi, M. Moskotin, I. Tretyakov, D. Yagodkin, S. Zhukov, T. Taniguchi, K. Watanabe, I. V. Grigorieva, M. Polini, G. N. Goltsman, A. K. Geim, G. Fedorov, Resonant terahertz detection using graphene plasmons. Nat. Commun. 9, 5392 (2018). 30568184 10.1038/s41467-018-07848-w PMC6300605

[65] R. M. Martin, L. Reining, D. M. Ceperley. *Interacting Electrons* (Cambridge Univ. Press, 2016).

[66] F. Hu, S. R. Das, Y. Luan, T.-F. Chung, Y. P. Chen, Z. Fei, Real-space imaging of the tailored plasmons in twisted bilayer graphene. Phys. Rev. Lett. 119, 247402 (2017). 29286712 10.1103/PhysRevLett.119.247402

[67] N. C. H. Hesp, I. Torre, D. Rodan-Legrain, P. Novelli, Y. Cao, S. Carr, S. Fang, P. Stepanov, D. Barcons-Ruiz, H. Herzig-Sheinfux, K. Watanabe, T. Taniguchi, D. K. Efetov, E. Kaxiras, P. Jarillo-Herrero, M. Polini, F. H. L. Koppens, Collective excitations in twisted bilayer graphene close to the magic angle. arXiv:1910.07893 [Preprint] [cond-mat.str-el] (17 October 2019).

[68] G. Baym, L. P. Kadanoff, Conservation laws and correlation functions. Phys. Rev. 124, 287–299 (1961).

[69] G. Baym, Self-consistent approximations in many-body systems. Phys. Rev. 127, 1391–1401 (1962).

[70] H. J. Monkhorst, J. D. Pack, Special points for Brillouin-zone integrations. Phys. Rev. B 13, 5188–5192 (1976).

