# Supplementary material for: Probing correlated states with plasmons
Source: Sci Adv. 2023 Apr 26;9(17):eadg3262. doi: 10.1126/sciadv.adg3262 (PMC12488045; doi:10.1126/sciadv.adg3262)
Supplement: Supplementary file 1 — Figs. S1 to S3 Table S1 Legends for movies S1 to S3 [file sciadv.adg3262_sm.pdf]

Science Advances
AAAS

Supplementary Materials for

Probing correlated states with plasmons

Michał Papaj *et al.*

Corresponding author: Michał Papaj, mpapaj@berkeley.edu;
Cyprian Lewandowski, clewandowski@magnet.fsu.edu

Sci. Adv. **9**, eadg3262 (2023)
DOI: 10.1126/sciadv.adg3262

The PDF file includes:

Figs. S1 to S3
Table S1
Legends for movies S1 to S3

Other Supplementary Material for this manuscript includes the following:

Movies S1 to S3

DIELECTRIC FUNCTION WITH LOCAL FIELD EFFECTS AND RELATION BETWEEN MACROSCOPIC AND MICROSCOPIC COEFFICIENTS

Here we briefly review the dielectric function with local field effects as introduced in the seminal works of Ref.42, 43. We start by discussing the form of the dielectric function with local field effects. As described in the main text, when calculated within the random phase approximation (RPA), the dielectric function matrix is given by[42–44]:

$$\varepsilon_{\mathbf{G}\mathbf{G}'}(\omega,\mathbf{q}) = \delta_{\mathbf{G}\mathbf{G}'} - T_{\mathbf{G}\mathbf{G}'}(\omega,\mathbf{q}) \tag{S1}$$

$$T_{\mathbf{G}\mathbf{G}'}(\omega,\mathbf{q}) = V_{\mathbf{q}+\mathbf{G}} \sum_{n,m,\mathbf{k}} \frac{f_0(\varepsilon_{n\mathbf{k}}) - f_0(\varepsilon_{m\mathbf{k}+\mathbf{q}})}{\omega + i0^+ + \varepsilon_{n\mathbf{k}} - \varepsilon_{m\mathbf{k}+\mathbf{q}}} \eta^{nm}_{\mathbf{q},\mathbf{G}}(\mathbf{k})^* \eta^{nm}_{\mathbf{q},\mathbf{G}'}(\mathbf{k}) \tag{S2}$$

where, as described in the main text, the Fourier transform of the Coulomb potential is given by $V_{\mathbf{q}} = \frac{2\pi e^2}{\kappa|\mathbf{q}|}$ (with κ being the dielectric constant of the surrounding insulating gate material), $f_0(\varepsilon) = (e^{\beta(\varepsilon-\mu)}+1)^{-1}$ (with μ the chemical potential and $\beta = 1/k_B T$ the inverse temperature). The state overlap $\eta^{nm}_{\mathbf{q},\mathbf{G}'}(\mathbf{k})$

$$\eta^{nm}_{\mathbf{q},\mathbf{G}}(\mathbf{k}) = \frac{1}{\Omega}\int_{\Omega} d^2\mathbf{r}\; u_{n\mathbf{k}}(\mathbf{r})^\dagger e^{-i\mathbf{G}\cdot\mathbf{r}} u_{m\mathbf{k}+\mathbf{q}}(\mathbf{r}) \tag{S3}$$

is evaluated using $u_{n\mathbf{k}}(\mathbf{r})$, the cell-periodic part of the Bloch wave function $\psi_{n\mathbf{k}}(\mathbf{r}) = u_{n\mathbf{k}}(\mathbf{r})e^{-i\mathbf{k}\cdot\mathbf{r}}$ for an eigenstate from band n with a Brillouin-zone momentum $\mathbf{k}$ and energy $\varepsilon_{n\mathbf{k}}$. The integral in Eq. (S3) is taken over the unit cell with real-space area Ω. The dispersion of plasmons is then given by the solution of a zero eigenvalue problem $\det \varepsilon_{\mathbf{G}\mathbf{G}'} = 0$ with the relevant eigenvectors corresponding to the real-space pattern of charge oscillations.

A crucial difference between moiré and conventional materials is the size of the effective unit cell. This difference particularly manifests when relating external electric field, with Fourier transform $\mathbf{E}(\mathbf{q})$ where $\mathbf{q}$ is unbounded, to the electric field inside the crystal, with a Fourier series components $\mathbf{E}_{\mathbf{G}}(\mathbf{q})$ where $\mathbf{q}$ is constrained to lie inside the 1st BZ. Physically this relation corresponds to the relation between microscopic and macroscopic quantities. To see this consider the analysis and discussion from Ref.42, which here we reproduce. We define a macroscopic quantity as the average of the corresponding microscopic quantity taken over a unit cell. We focus here on a function $f(\mathbf{r})$ where $\mathbf{r} = \mathbf{R} + \mathbf{u}$ is a vector in the microscopic space of the crystal. We denote its macroscopic average by $\tilde{f}(\mathbf{R})$, where the bar indicates an average taken over the unit cell whose location is given by the macroscopic position variable $\mathbf{R}$. Here $\mathbf{u}$ is a vector defined within the crystal's unit cell. We then have

$$\tilde{f}(\mathbf{R}) = \frac{1}{\Omega}\sum_{\mathbf{q},\mathbf{G}} f_{\mathbf{G}}(\mathbf{q}) \int_{\Omega} d^2\mathbf{r} e^{-i(\mathbf{q}+\mathbf{G})\cdot\mathbf{r}} = \sum_{\mathbf{q},\mathbf{G}} \alpha_{\mathbf{G}}(\mathbf{q}) f_{\mathbf{G}}(\mathbf{q}) e^{-i\mathbf{q}\cdot\mathbf{R}}\,, \tag{S4}$$

where the coefficients $\alpha_{\mathbf{G}}(\mathbf{q})$ are given by

$$\alpha_{\mathbf{G}}(\mathbf{q}) = \frac{1}{\Omega}\int_{\Omega} d^2\mathbf{u}\, e^{-i(\mathbf{q}+\mathbf{G})\cdot\mathbf{u}} \tag{S5}$$

At the same time a macroscopic Fourier transform of $\tilde{f}(\mathbf{R})$ is given by

$$\tilde{f}(\mathbf{R}) = \int d^2\mathbf{q}\, \tilde{f}(\mathbf{q}) e^{i\mathbf{q}\cdot\mathbf{R}} \tag{S6}$$

giving a relation between macroscopic and microscopic coefficients

$$\tilde{f}(\mathbf{q}) = \sum_{\mathbf{G}} \alpha_{\mathbf{G}}(\mathbf{q}) f_{\mathbf{G}}(\mathbf{q})\,. \tag{S7}$$

If the unit cell is small (or equivalently $\mathbf{q} \to 0$), then the coefficients $\alpha_{\mathbf{G}}(\mathbf{q}) \approx \delta_{\mathbf{G},0}$[42]. In the context of moiré systems, however, this relation does not hold, and $\alpha_{\mathbf{G}}(\mathbf{q})$ has finite support for non-zero $\mathbf{G}$. This physics is what allows for a non-trivial dependence of the field in the crystal $\mathbf{E}_{\mathbf{G}}(\mathbf{q})$ to manifest in macroscopic quantity $\mathbf{E}(\mathbf{q})$.

THE 2×2 PLASMON FOLDING MODEL

In this section, we derive the effective form of the dielectric function from Eq. 10 in the main text used to describe plasmon folding analytically.

As argued in the main text, to demonstrate the mechanism of plasmon folding, we focus on the behavior of $\varepsilon_{\mathbf{GG}'}(\omega, \mathbf{q})$ near the BZ edge at $\mathbf{q}_M = -\mathbf{G_1}/2$ (chosen to be the M point of the hexagonal BZ) for the case of $\nu = 2/3$ filling shown in Fig. 1(c). Near the momentum $\mathbf{q}_M$, only the matrix entries corresponding to momenta $\mathbf{G} = 0, \mathbf{G_1}$ contribute to the leading order in the $\varepsilon_{\mathbf{GG}'}(\omega, \mathbf{q})$ due to the Coulomb prefactor $1/|\mathbf{q} + \mathbf{G}|$. The resulting dielectric function matrix has the structure of Eq. (9) from the main text:

$$\varepsilon_{\mathbf{GG}'}(\omega, \mathbf{q}) = \begin{bmatrix} 1 - T_{00}(\omega, \mathbf{q}) & -T_{0\mathbf{G}_1}(\omega, \mathbf{q}) \\ -T_{\mathbf{G}_1 0}(\omega, \mathbf{q}) & 1 - T_{\mathbf{G}_1\mathbf{G}_1}(\omega, \mathbf{q}) \end{bmatrix}. \tag{S8}$$

We now proceed to derive the form of Eq. (10) from the main text.

The plasmons discussed in the main text systems rise over the particle-hole continua and emerge in the energy gap. To understand their behavior analytically, we focus on the frequency range ω that lies in the energy gap between interband transitions. As such we can then split the sum over bands n, m in the function $T_{\mathbf{GG}'}(\omega, \mathbf{q})$ from Eq. (S1) into two distinct parts:

$$T_{\mathbf{GG}'}(\omega, \mathbf{q}) = \sum_{m,n}{}' \cdots + \sum_{m,n}{}'' \cdots \tag{S9}$$

Here the first sum $\sum'_{m,n}$ ranges over states such that $|\varepsilon_{n\mathbf{k}} - \varepsilon_{m\mathbf{k}+\mathbf{q}}| < \omega$ and the second sum $\sum''_{m,n}$ ranges over states $|\varepsilon_{n\mathbf{k}} - \varepsilon_{m\mathbf{k}+\mathbf{q}}| > \omega$, where ω is the relevant frequency range in the energy gap between particle-hole continua where the plasmon will appear.

To proceed further, let us also assume that only two bands $n, m = \pm 1$ exist in the model separated by an energy scale Δ. One band $(-)$ is occupied, and the other band $(+)$ is unoccupied, with the chemical potential in the gap between them. In such a case, a plasmon will appear from interband transitions at energies higher than the energies of band $+$. Since no other bands exist in this toy model, the $\sum''_{m,n}$ vanishes. If there were higher bands, their role would be to soften the resulting plasmon dispersion as discussed in Ref. 57. This assumption does not modify the qualitative behavior of plasmon folding. For the $\sum'_{m,n}$ we have

$$T_{\mathbf{GG}'}(\omega, \mathbf{q}) \approx V_{\mathbf{q}+\mathbf{G}} \quad \sum_{\mathbf{k}} \frac{-1}{\omega + \Delta} \eta^{+-}_{\mathbf{q},\mathbf{G}}(\mathbf{k})^* \eta^{+-}_{\mathbf{q},\mathbf{G}'}(\mathbf{k}) + \sum_{\mathbf{k}} \frac{1}{\omega - \Delta} \eta^{-+}_{\mathbf{q},\mathbf{G}}(\mathbf{k})^* \eta^{-+}_{\mathbf{q},\mathbf{G}'}(\mathbf{k}) \Big) \approx \tag{S10}$$

$$\approx \frac{V_{\mathbf{q}+\mathbf{G}}}{\omega^2 - \Delta^2} \sum_{\mathbf{k}} \Delta \left(\eta^{+-}_{\mathbf{q},\mathbf{G}}(\mathbf{k})^* \eta^{+-}_{\mathbf{q},\mathbf{G}'}(\mathbf{k}) + \eta^{-+}_{\mathbf{q},\mathbf{G}}(\mathbf{k})^* \eta^{-+}_{\mathbf{q},\mathbf{G}'}(\mathbf{k}) \right) + \omega \left(\eta^{-+}_{\mathbf{q},\mathbf{G}}(\mathbf{k})^* \eta^{-+}_{\mathbf{q},\mathbf{G}'}(\mathbf{k}) - \eta^{+-}_{\mathbf{q},\mathbf{G}}(\mathbf{k})^* \eta^{+-}_{\mathbf{q},\mathbf{G}'}(\mathbf{k}) + \right) \tag{S11}$$

$$\approx \frac{V_{\mathbf{q}+\mathbf{G}}}{\omega^2 - \Delta^2} \sum_{\mathbf{k}} \Delta \left(\eta^{+-}_{\mathbf{q},\mathbf{G}}(\mathbf{k})^* \eta^{+-}_{\mathbf{q},\mathbf{G}'}(\mathbf{k}) + \eta^{-+}_{\mathbf{q},\mathbf{G}}(\mathbf{k})^* \eta^{-+}_{\mathbf{q},\mathbf{G}'}(\mathbf{k}) \right) \tag{S12}$$

In arriving at the first line, we approximated the energy difference between the bands as $\pm\Delta$ and explicitly carried out the sum of $m, n = \pm$. We assume the $T = 0$ limit in which case $f_0(\varepsilon_{-\mathbf{k}}) = 1$ and $f_0(\varepsilon_{+\mathbf{k}}) = 0$. In the second line, we combined the two sums, and in the third line, we dropped the term odd in ω as it vanished by the time-reversal symmetry.

Carrying this analysis for all entries of the Eq.(S8), we arrive at Eq. (10) of the main text

$$\varepsilon_{\mathbf{GG}'}(\omega, \mathbf{q}) \approx \begin{bmatrix} 1 - \frac{A(\mathbf{q})}{\omega^2 - \Delta^2} & -\frac{C(\mathbf{q})}{\omega^2 - \Delta^2} \\ -\frac{D(\mathbf{q})}{\omega^2 - \Delta^2} & 1 - \frac{B(\mathbf{q})}{\omega^2 - \Delta^2} \end{bmatrix} \tag{S13}$$

where the coefficients $A(\mathbf{q})$,$B(\mathbf{q})$,$C(\mathbf{q})$,$D(\mathbf{q})$ are given by:

$$A(\mathbf{q}) = V_{\mathbf{q}} \sum_{\mathbf{k}} \Delta \left(\eta^{+-}_{\mathbf{q},0}(\mathbf{k})^* \eta^{+-}_{\mathbf{q},0}(\mathbf{k}) + \eta^{-+}_{\mathbf{q},\mathbf{G}}(\mathbf{k})^* \eta^{-+}_{\mathbf{q},\mathbf{G}'}(\mathbf{k}) \right) \tag{S14}$$

$$B(\mathbf{q}) = V_{\mathbf{q}+\mathbf{G_1}} \sum_{\mathbf{k}} \Delta \left(\eta^{+-}_{\mathbf{q},\mathbf{G_1}}(\mathbf{k})^* \eta^{+-}_{\mathbf{q},\mathbf{G_1}}(\mathbf{k}) + \eta^{-+}_{\mathbf{q},\mathbf{G_1}}(\mathbf{k})^* \eta^{-+}_{\mathbf{q},\mathbf{G_1}}(\mathbf{k}) \right) \tag{S15}$$

$$C(\mathbf{q}) = V_{\mathbf{q}} \sum_{\mathbf{k}} \Delta \left(\eta^{+-}_{\mathbf{q},0}(\mathbf{k})^* \eta^{+-}_{\mathbf{q},\mathbf{G_1}}(\mathbf{k}) + \eta^{-+}_{\mathbf{q},0}(\mathbf{k})^* \eta^{-+}_{\mathbf{q},\mathbf{G_1}}(\mathbf{k}) \right) \tag{S16}$$

$$D(\mathbf{q}) = V_{\mathbf{q}+\mathbf{G_1}} \sum_{\mathbf{k}} \Delta \left(\eta^{+-}_{\mathbf{q},\mathbf{G_1}}(\mathbf{k})^* \eta^{+-}_{\mathbf{q},0}(\mathbf{k}) + \eta^{-+}_{\mathbf{q},\mathbf{G_1}}(\mathbf{k})^* \eta^{-+}_{\mathbf{q},0}(\mathbf{k}) \right) \tag{S17}$$

We can approximately evaluate these coefficients in Eq.(S14) using the Thomas-Reiche-Kuhn sum rule. Specifically, we have:

$$\sum_{\mathbf{k}} \Delta \left(\eta^{+-}_{\mathbf{q},\mathbf{G}}(\mathbf{k})^* \eta^{+-}_{\mathbf{q},\mathbf{G}'}(\mathbf{k}) + \eta^{-+}_{\mathbf{q},\mathbf{G}}(\mathbf{k})^* \eta^{-+}_{\mathbf{q},\mathbf{G}'}(\mathbf{k}) \right) \approx \frac{n}{m} (\mathbf{q}+\mathbf{G}) \cdot (\mathbf{q}+\mathbf{G}') \bar{\eta}^{+}_{\mathbf{G}} \bar{\eta}^{-}_{\mathbf{G}'} \tag{S18}$$

Here n/m corresponds to the ratio of charge density to electron mass and characterizes the scale for electron-electron interactions. The numbers $\bar{\eta}^{+}_{\mathbf{G}}$, $\bar{\eta}^{-}_{\mathbf{G}'}$ characterize how the average (over BZ) spectral weight of Bloch states for a band $\pm$ vanishes with $\mathbf{G}$. In general, for continuum models, we expect it to be an exponential decay, i.e., $\bar{\eta}^{+}_{\mathbf{G}}, \bar{\eta}^{-}_{\mathbf{G}} \propto \exp\left(-\xi^2 \mathbf{G}^2\right)$ with the characteristic decay length ξ set by the Wannier-function spread (See Ref.56). Based on the Eq. (S18), for the plot of Fig.1(b) we therefore use

$$A(\mathbf{q}) = \alpha q \tag{S19}$$

$$B(\mathbf{q}) = \alpha(2\pi - q) \tag{S20}$$

$$C(\mathbf{q}) D(\mathbf{q}) = \alpha^2 \eta q (2\pi - q)\,, \tag{S21}$$

where $\alpha \propto n/m = 0.15$ set the scale of electron-electron to kinetic energy, $\eta = 0.1$ controls the Wannier-function spread as explained above, and $\mathbf{G} = 2\pi$ in natural units. Note that for the plasmon dispersion of Eq. (11), functions $C(\mathbf{q})$ and $D(\mathbf{q})$ only enter as a product.

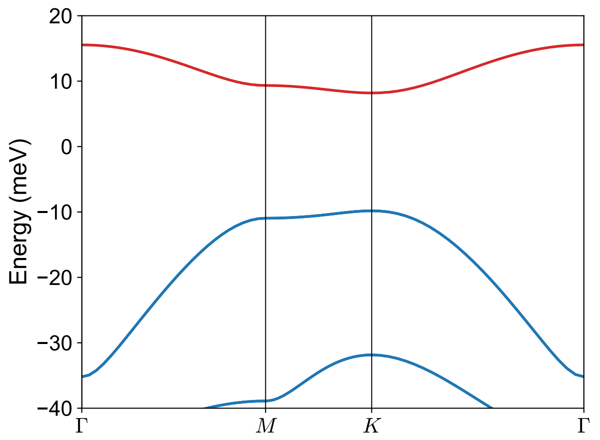

FIG. S1. **The moiré band structure of WSe_2/WS_2 heterobilayer.** The bands are obtained through the diagonalization of Hamiltonian from Eq. (4) from the main text, with the parameters as given there. The top-most valence moiré flat band, which is the source of the correlated phases, is indicated in red.

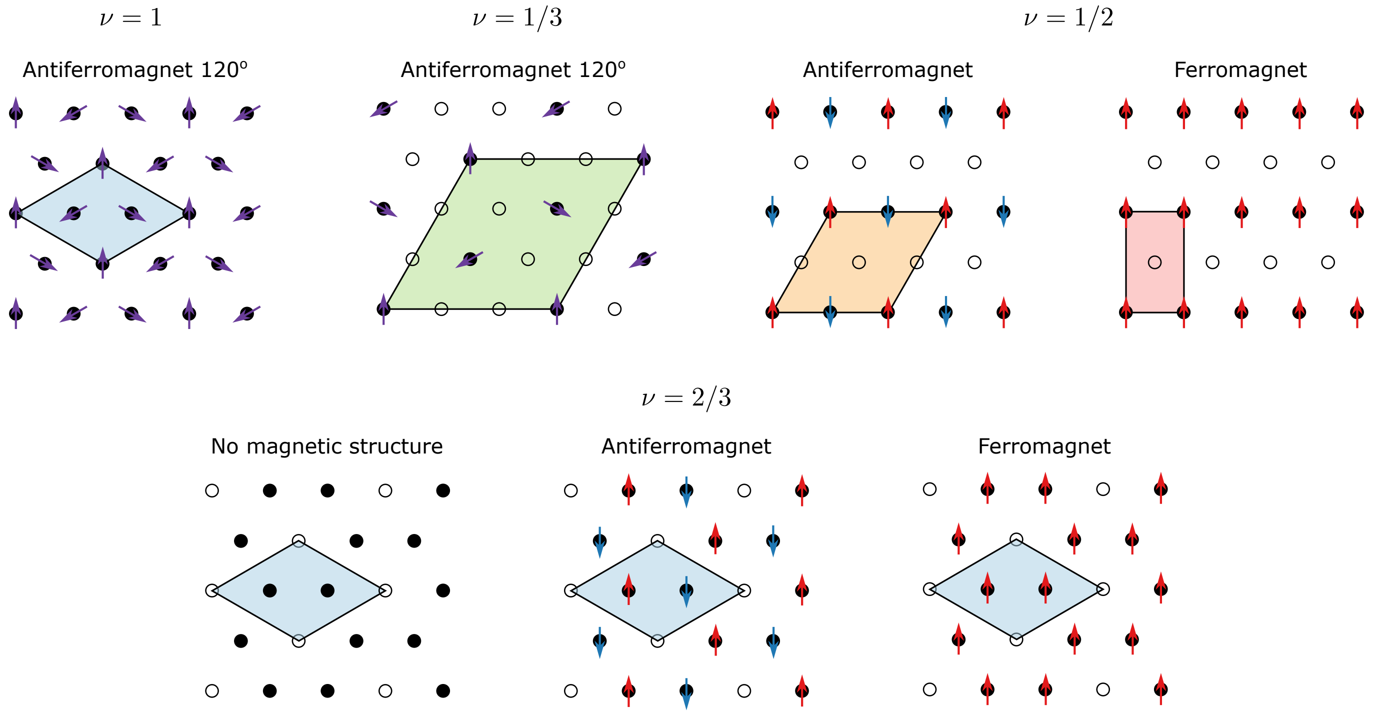

FIG. S2. **Structure of the studied ground states at various fillings.** Depending on the filling fraction ν we consider different ground states with varying magnetic structure. In each case, the state is a generalized Wigner crystal with the unit cell enlarged as compared to the moiré unit cell. Blue, green, orange, and red unit cells are 3, 9, 4, 2 times larger than moiré cell, respectively. The magnetic structure is shown via arrows on the filled Wigner crystal sites, with blue and red pointing along z direction perpendicular to the plane, and purple arrows located within the $x-y$ plane.

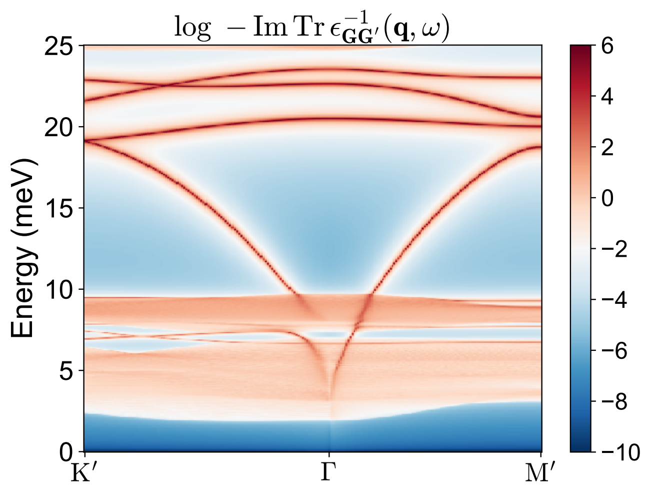

FIG. S3. **The trace of the electron loss function at $\nu = 1/2$ filling in antiferromagnetic state.** Multiple plasmon branches are present due to local field effects. However, in contrast to the analogous figure in the main text (Fig. 2), for a different (inequivalent) choice of M point in the Brillouin zone, the plasmon bands become gapped as the relevant off-diagonal elements of $\varepsilon_{\mathbf{GG}'}$ are not vanishing.

Filling fraction ν	1	1/3	1/2		2/3		
Magnetic state	AF 120°	AF 120°	AF	FM	No spin	AFM	FM
Lattice vectors $\mathbf{a}_{W1}, \mathbf{a}_{W2}$	$\mathbf{a}_1 + \mathbf{a}_2$, $2\mathbf{a}_1 - \mathbf{a}_2$	$3\mathbf{a}_1$, $3\mathbf{a}_2$	$2\mathbf{a}_1, 2\mathbf{a}_2$	$2\mathbf{a}_1, \mathbf{a}_2$	$\mathbf{a}_1 + \mathbf{a}_2$, $2\mathbf{a}_1 - \mathbf{a}_2$		
Unit cell enlargement	3	9	4	2	3		
Basis vectors τ	$\mathbf{0}, \mathbf{a}_1, 2\mathbf{a}_1$	$\mathbf{0}$, $\mathbf{a}_1 + \mathbf{a}_2$, $2\mathbf{a}_1 + 2\mathbf{a}_2$	$\mathbf{0}$, $\mathbf{a}_2$	$\mathbf{0}$	$\mathbf{a}_1, 2\mathbf{a}_1$		
V_C/V_0	0	0	0	0	-0.284	0	0
V_B/V_0	0.08	0.062	0.0442	0.07	0	0.0446	0.0958
Estimated gap (meV)	7.5	3.5	1.9		2.9		

TABLE S1. **Parameters for the effective models.** Each of the seven different ground states has a separate set of parameters that aim to reproduce experimentally observed gaps. The magnetic structures are visualized in Fig. S2. Lattice and basis vectors of the generalized Wigner crystal are expressed in terms of moiré lattice vectors from Eq. (13). Strengths of effective Wigner crystal potential are given in terms of moiré potential strength $V_0 = 15\,\mathrm{meV}$.

Movie S1. **Evolution of the folded plasmon spectrum as off-diagonal terms in $\varepsilon_{\mathbf{GG'}}$ are introduced.** The movie follows the evolution of an energy loss function as the off-diagonal terms of $\varepsilon_{\mathbf{GG'}}$ are switched on, with γ defined as $\varepsilon_{\mathbf{GG'}} = \varepsilon_{\mathbf{GG'}}\delta_{\mathbf{GG'}} + \gamma(1-\delta_{\mathbf{GG'}})\varepsilon_{\mathbf{GG'}}$. The calculation is performed at filling fraction $\nu = 2/3$ for a ground state with only the Hartree-like potential. Initially, a large number of plasmon modes are visible as the results of zeros in the diagonal elements of $\varepsilon_{\mathbf{GG'}}$. However, the increase in the coefficient γ leads to a stronger impact of the off-diagonal elements, which results in all but three plasmon bands being pushed into the particle-hole continuum. The number of remaining plasmons equals the unit cell enlargement factor of the generalized Wigner crystal.

Movie S2. **Evolution of the plasmon spectrum with increasing Hartree-like potential.** The movie shows how the energy loss function changes when constant electron filling $\nu = 2/3$ is maintained as the fitting parameter V_C of the Hartree-like potential from Eq. (7) of the main text is increased. The starting point is the complete absence of Hartree potential ($V_C = 0$, corresponding to the right panel of Fig.1c), and the endpoint is the value that reproduces experimentally observed gap ($V_C = 0.0284V_0$, the left panel of Fig.1c). Opening of the correlated gap can be observed as the particle-hole continuum is raised, and gaps open between the plasmonic bands.

Movie S3. **Evolution of the unfolded plasmon spectrum as off-diagonal terms in $\varepsilon_{\mathbf{GG'}}$ are introduced.** The movie follows the evolution of an energy loss function as the off-diagonal terms of $\varepsilon_{\mathbf{GG'}}$ are switched on, with γ defined as $\varepsilon_{\mathbf{GG'}} = \varepsilon_{\mathbf{GG'}}\delta_{\mathbf{GG'}} + \gamma(1-\delta_{\mathbf{GG'}})\varepsilon_{\mathbf{GG'}}$. The calculation is performed at the electron density corresponding to filling fraction $\nu = 2/3$, but without forming any Wigner crystal (no Hartree or effective magnetic field included). Initially, a large number of plasmon modes are visible as the results of zeros in the diagonal elements of $\varepsilon_{\mathbf{GG'}}$. However, as γ increases, all plasmon modes except one are pushed into the particle-hole continuum.
